# Growth improvement following antiretroviral therapy initiation in children with perinatally-acquired HIV diagnosed in older childhood in Zimbabwe: a prospective cohort study

**DOI:** 10.1186/s12887-022-03466-0

**Published:** 2022-07-25

**Authors:** Victoria Simms, Grace McHugh, Ethel Dauya, Tsitsi Bandason, Hilda Mujuru, Kusum Nathoo, Shungu Munyati, Helen A. Weiss, Rashida A. Ferrand

**Affiliations:** 1 MRC International Statistics & Epidemiology Group, London School of Hygiene & Tropical Medicine, London, UK; 2 The Health Research Unit Zimbabwe, Harare, Zimbabwe; 3 Department of Paediatrics, University of Zimbabwe, Harare, Zimbabwe; 4 Clinical Research Department, London School of Hygiene & Tropical Medicine, London, UK

**Keywords:** Adolescents, Children, Growth, Puberty, Stunting

## Abstract

**Background:**

Children who initiate antiretroviral therapy (ART) before age 5 years can recover height and weight compared to uninfected peers, but growth outcomes are unknown for children initiating ART at older ages. We investigated factors associated with growth failure at ART initiation and modelled growth by age on ART.

**Methods:**

We conducted secondary analysis of cohort of children aged 6–15 years late-diagnosed with HIV in Harare, Zimbabwe, with entry at ART initiation in 2013–2015.

Factors associated with height-for-age (HAZ), weight-for-age (WAZ) and BMI-for-age (BAZ) z-scores <− 2 (stunting, underweight and wasting respectively) at ART initiation were assessed using multivariable logistic regression. These outcomes were compared at ART initiation and 12 month follow-up using paired t-tests. HAZ and BAZ were modelled using restricted cubic splines.

**Results:**

Participants (*N* = 302; 51.6% female; median age 11 years) were followed for a median of 16.6 months (IQR 11.0–19.8). At ART initiation 34.8% were stunted, 34.5% underweight and 15.1% wasted. Stunting was associated with age ≥ 12 years, CD4 count < 200 cells/μl, tuberculosis (TB) history and history of hospitalisation. Underweight was associated with older age, male sex and TB history, and wasting was associated with older age, TB history and hospitalisation. One year post-initiation, t-tests showed increased WAZ (*p* = 0.007) and BAZ (*p* = 0.004), but no evidence of changed HAZ (*p* = 0.85). Modelling showed that HAZ and BAZ decreased in early adolescence for boys on ART, but not girls.

**Conclusion:**

Stunting and underweight were prevalent at ART initiation among late-diagnosed children, and HAZ did not improve after 1 year. Adolescent boys with perinatally acquired HIV and late diagnosis are particularly at risk of growth failure in puberty.

**Supplementary Information:**

The online version contains supplementary material available at 10.1186/s12887-022-03466-0.

## Introduction

Perinatal HIV acquisition affects children’s growth through multiple pathways, including clinical factors such as reduced absorption of nutrients due to HIV enteropathy, altered metabolic rates, chronic inflammation and social factors such as poverty, poor diet, emotional deprivation and stress [1, 2]. Growth failure including stunting and pubertal delay is common among children with perinatally acquired HIV, and more marked among those in resource-limited settings [3, 4]. This may be partly due to delayed initiation of antiretroviral therapy (ART). Treatment guidelines for resource-limited settings have long recommended immediate ART initiation for children aged less than 2 years, but guidelines for children aged 2 and older recommended ART initiation based on disease and/or immunological stage until 2016 when immediate ART was recommended for all regardless of age and disease stage [3]. Immediate ART initiation has many advantages [4], but many children have initiated ART in older childhood or adolescence due to delayed HIV diagnosis [5], particularly in sub-Saharan Africa where 90% of the world’s children with HIV live [6]. In a global cohort analysis conducted in 2018 by the CIPHER Global Cohort Collaboration, the median age of ART initiation among children in sub-Saharan Africa was 7.9 years, compared to < 1 year old in high-income countries [7]. In this meta-analysis, 30,296 children living with perinatally acquired HIV and diagnosed under 10 years of age in sub-Saharan Africa had a median height-for-age z-score (HAZ) of − 2.0 when first diagnosed (median age 7.1 years, IQR 5.3–8.6) and − 1.8 at the end of follow-up (median age 12.1, IQR 10.9–13.8) [7].

Children who initiate ART aged under 3 years old can experience rapid growth, and catch up with their HIV negative peers [8]. However, there is little evidence that children who initiate ART at older ages achieve catch-up growth. A multi-regional analysis of 19 cohorts (including some CIPHER cohorts) and 20,576 children, mainly from southern Africa, found that children aged under 10 years old gained height-for-age after ART initiation, especially if initiation was immediately following diagnosis rather than later, based on CD4 count criteria [9]. Among adolescents aged 10–16 years, the timing of ART initiation appeared to have little effect on HAZ [9].

The aim of this study was to investigate the factors associated with growth failure at ART initiation in a cohort of children and adolescents diagnosed with HIV aged 6–15 years in Zimbabwe, and to model growth following ART initiation.

## Methods

Between January 2013 and December 2014, routine opt-out provider-initiated HIV testing and counselling was implemented in seven primary care clinics in southwest Harare, Zimbabwe for all attendees aged 6–15 years. Routine opt-out testing using the nationally recommended HIV antibody testing algorithm was performed for every child attending for any reason who had a caregiver able to give consent, unless the child had a documented HIV negative test within the previous 6 months or an HIV positive test [10]. All children newly-diagnosed with HIV who resided in Harare were enrolled into a cohort study, with caregiver consent and child assent. The methods of the cohort study have been explained in detail elsewhere [11]. In brief, children diagnosed with HIV infection were registered for HIV care at the same clinic where they were diagnosed with caregiver consent and participant assent. Nurse-led HIV care was provided with physician supervision according to national guidelines. At the initial assessment visit, past clinical history was ascertained and HIV staging, CD4 count testing and TB screening was performed. The schedule for follow up was based on national guidelines, with visits at 2 and 6 weeks post ART commencement and three monthly subsequently. At each visit, a standard proforma was used to record symptoms, side-effects of ART and contact with health services. Participants not eligible for ART at baseline underwent a 3-monthly symptom-based review and examination to reassess ART eligibility. Eligibility for ART initiation was determined following national guidelines, which in 2013 were a CD4 count< 350 cells/μl. In February 2014 national guidelines were updated to initiate ART at < 500 cells/μl.

Three trained research nurses collected the data. Height and weight were recorded at enrolment and every 3 months up to 18 months of follow-up. Height was measured with a SECA stadiometer and weight with a digital scale. BMI (kg/m^2^) was calculated from weight and height. Z-scores were calculated using the 1990 UK reference populations [12]. Z-scores > 5 or < − 5 were excluded as outliers. Stunting at ART initiation was defined as a height-for-age z-score (HAZ) below − 2, being underweight as a weight-for-age z-score (WAZ) below − 2, and wasting was defined as a BMI-for-age z-score (BAZ) below − 2. Pubertal development was assessed using Tanner Staging. Girls were defined as having delayed puberty if they were at Tanner breast development stage 1 for any measurements when aged > 13 years. Boys were defined as delayed puberty if they were at Tanner stage 1 for testes development at any measurement aged > 14 years. Route of HIV acquisition was determined from the parents’ HIV status if known, self-report of sexual debut or likely parenteral routes of transmission (blood transfusion. Surgery etc) and clinical history (history of chronic illness since early childhood).

For this paper, ART initiation was used as the cohort entry point. Height, weight and CD4 count at ART initiation were defined as the measures taken at the closest visit to ART initiation, within a window period from 100 days prior up to 30 days post ART initiation. Participants who did not have a measurement within this window were excluded. Z-scores of HAZ, WAZ and BAZ were coded into four categories, <− 3, − 3 to <− 2, − 2 to <− 1 and ≥ − 1. CD4 count was categorised as < 200, 200–349, 350–499 and ≥ 500 cells/ml. Characteristics of the cohort population were described at ART initiation and at 1 year post-initiation (defined as the record closest to 12 months post-initiation, within the 9–15 months period). Paired t-tests were used to compare HAZ, WAZ and BAZ at ART initiation and 1 year later.

Association with stunting, underweight and wasting at ART initiation was investigated using univariable logistic regression to estimate odds ratios (ORs) for the following variables: death of a parent, history of hospital admission, TB history, and CD4 count (< 200 or ≥ 200 cells/ml). Variables associated with the outcome in univariable analysis (*p*-value < 0.1 using likelihood ratio tests) were included in a multivariable model. Age and sex were included a priori.

A restricted cubic spline [13] with 4 knots was used to model HAZ over age in months from ART initiation onwards, in a multilevel mixed-effects linear regression model adjusting for the a priori selected 4-category ordinal variables: age at ART initiation, CD4 count at ART initiation, and HAZ at ART initiation. An interaction between sex and spline terms was included and a likelihood ratio test was used to compare models with and without the interaction. The same process was used to model BAZ. WAZ was not modelled due to the amount of within-participant fluctuation. Median-spline graphs with 8 bands of the fitted predictions and 95% predicted intervals were laid over the individual trajectories. Predicted values of HAZ and BAZ were obtained from the model coefficients, over a range of values of key variables. For these predicted values age was input as exact age in years (i.e. 6 years and 0 days, 7 years and 0 days etc) up to 18 years.

## Results

**Fig. 1:**
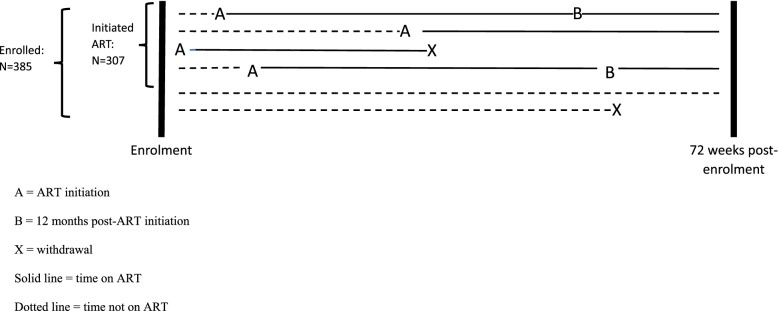
Diagram of enrolment, ART initiation and follow-up. A = ART initiation. B = 12 months post-ART initiation. X = withdrawal. Solid line = time on ART. Dotted line = time not on ART

A total of 385 participants newly-diagnosed with HIV were enrolled into the parent cohort study [9], 307 of whom (79.7%) initiated ART (Fig. 1). At enrolment the 307 participants were aged 6–16 (median 11) years and 45.0% (*N* = 138) had WHO stage 3–4 HIV disease. Nurses determined that the most likely route of HIV acquisition for 97% of these children (*N* = 297) was perinatal transmission. Participants were aged 6–17 (median 11) years at ART initiation. The main reason for participants not initiating ART was non-eligibility according to the national guidelines at the time (*n* = 68; 87.2%) and 10 did not initiate for other reasons. Follow-up on ART is shown in Fig. 1, with 6 imaginary participants representing the various possible follow-up pathways. Out of 307 participants who initiated ART (A), 220 had a 12-month follow-up (B), while the remaining 87 were either lost to follow-up before the end of the 72-week observation period or initiated ART less than 12 months before its end. The median duration of follow-up from ART initiation to the final anthropometry record was 498 days (IQR 329–593), up to 950 days post-enrolment. Participants had between 1 and 14 records each (median 7, IQR 4–9). For 33 participants (10.7%) follow-up ended with transfer to another clinic, 12 (3.9%) died, 2 (0.7%) moved away, 6 (2.0%) were lost to follow-up and 254 (82.7%) reached the end of follow-up in the parent study. Six of the 12 deaths occurred within 3 months of ART initiation. The specific ART regimen was recorded for 260 participants (84.7%), of whom 129 (49.6%) were taking a tenofovir (TDF)-based regimen, 121 (46.5%) a zidovudine (AZT)-based regimen, and 10 were taking stavudine (d4T). Of these 10 participants, 8 switched to another regimen during follow-up, 1 transferred to another clinic and 1 had an unknown regimen after initiation. Most participants (92.5%, *n* = 284) were in school, although 8 of the 30 participants aged ≥15 years (26.7%) no longer attended school. Just over half (*n* = 174, 56.7%) of participants were cared for by a parent, 60 (19.5%) by an aunt or uncle, 52 (16.9%) by a grandparent, 17 (5.5%) by another relative and 4 (1.3%) by an institution. Since birth, 174 (57.4%) had changed primary caregiver at least once.

Of the 307 cohort participants, 302 (98.4%) had a valid HAZ measurement (i.e. between − 5 and 5) taken during the ART initiation visit window. WAZ was measured and valid for 296 (96.4%) participants, and BAZ for 298 participants (97.1%). A total of 301 participants had height measurements at least once post-ART initiation.

### Risk factors for stunting, underweight and wasting at ART initiation

At ART initiation, 105 (34.8%) participants were stunted (HAZ < -2) of whom 36 (34.3%) had severe stunting (HAZ < -3) (Table 1). About one-third of participants (34.5%, *N* = 102) were underweight (WAZ < -2), and 45 (44.1%) of these were severely underweight (WAZ < -3). The prevalence of wasting (BAZ < -2) was 15.1%, of whom 38% had a BAZ < -3. Boys had a higher prevalence than girls of severe stunting (17.1% vs 7.1%, *p* = 0.007), severe underweight (20.6% vs 10.3%, *p* = 0.01) and severe wasting (8.4% vs 3.2%, *p* = 0.05). Prevalence of these conditions using the z-score < − 2 definition was also higher among boys than girls, but the difference was only statistically significant for underweight (41.8% vs 27.7%, *p* = 0.01). At ART initiation 23.8% of participants had a history of hospital admission and 5.8% had a history of TB.

**Table 1:** Descriptive characteristics of participants at ART initiation and 1 year post-initiation

	ART initiation			1 year post-initiation		
	Male, n(%)	Female, n(%)	Total, n(%)	Male, n(%)	Female, n(%)	Total, n(%)
N	146	156	302	114	112	226
Age (years)						
6–8	34 (23.2)	26 (16.7)	60 (19.9)	15 (13.2)	7 (6.3)	22 (9.7)
9–11	46 (31.5)	61 (39.1)	107 (35.4)	34 (29.8)	36 (32.1)	70 (31.0)
12–13	34 (23.3)	35 (22.4)	69 (22.9)	23 (20.2)	33 (29.5)	56 (24.8)
14–17	32 (21.9)	33 (21.8)	66 (21.9)	42 (36.8)	36 (32.1)	78 (34.5)
HAZ						
Mean (SD) Range	−1.80 (1.18) − 4.5 to 1.3	− 1.52 (1.08) −5.0 to 1.4	−1.65 (1.13) − 5.0 to 1.4	−1.76 (1.12) − 4.2 to 1.1	−1.53 (1.08) − 4.6 to 0.8	−1.65 (1.10) − 4.6 to 1.1
Below − 3	25 (17.1)	11 (7.1)	36 (11.9)	20 (17.5)	11 (9.8)	31 (13.7)
− 3 to <− 2	29 (19.9)	40 (25.6)	69 (22.9)	20 (17.5)	23 (20.5)	43 (19.0)
−2 to <−1	55 (37.7)	57 (36.5)	112 (37.1)	45 (39.5)	42 (37.5)	87 (38.5)
−1 and above	37 (25.3)	48 (30.1)	85 (28.2)	29 (25.4)	36 (32.1)	65 (28.8)
Stunted (<−2)	54 (37.0)	51 (32.7)	105 (34.8)	40 (35.1)	34 (30.4)	74 (32.7)
WAZ						
Mean (SD) range	−1.81 (1.22) −4.3 to 0.4	−1.39 (1.11) − 4.5 to 1.0	−1.59 (1.18) − 4.5 to 1.0	−1.72 (1.13) − 4.5 to 0.7	−1.17 (1.07) − 4.2 to 0.9	−1.45 (1.13) − 4.5 to 0.9
Below −3	29 (20.6)	16 (10.3)	45 (15.2)	18 (16.1)	7 (6.4)	25 (11.3)
−3 to <− 2	30 (21.3)	27 (17.4)	57 (19.3)	23 (20.5)	15 (13.8)	38 (17.2)
−2 to <−1	39 (27.7)	53 (34.2)	92 (31.1)	38 (33.9)	37 (33.9)	75 (33.9)
−1 and above	43 (30.5)	59 (38.1)	102 (34.5)	33 (29.5)	50 (45.9)	83 (37.6)
Missing	5	1	6	2	3	5
Underweight (<−2)	59 (41.8)	43 (27.7)	102 (34.5)	41 (36.6)	22 (18.5)	63 (28.5)
BAZ						
Mean (SD) range	−1.11 (1.17) −4.6 to 1.4	−0.72 (1.06) −4.3 to 1.3	−0.91 (1.12) − 4.6 to 1.4	−0.97 (1.00) −3.6 to 0.8	−0.42 (0.99) − 2.9 to 1.6	− 0.70 (1.03) − 3.6 to 1.6
Below − 3	12 (8.4)	5 (3.2)	17 (5.7)	6 (5.3)	0	6 (2.7)
−3 to <−2	14 (9.8)	14 (9.0)	28 (9.4)	11 (9.7)	9 (8.3)	20 (9.0)
−2 to <−1	48 (33.6)	35 (22.6)	83 (27.9)	34 (30.1)	17 (15.6)	51 (23.0)
−1 and above	69 (48.3)	101 (65.2)	170 (57.1)	62 (54.9)	83 (76.2)	145 (65.3)
Missing	3	1	4	1	3	4
Wasted (<−2)	26 (18.2)	19 (12.3)	45 (15.1)	17 (15.0)	9 (8.3)	26 (11.7)
CD4 count						
< 200	41 (30.4)	47 (32.2)	88 (31.3)	10 (14.7)	12 (16.4)	22 (15.6)
200–349	44 (32.6)	46 (31.5)	90 (32.0)	4 (5.9)	13 (17.8)	17 (12.1)
350–499	35 (25.9)	36 (24.7)	71 (25.3)	16 (23.5)	7 (9.6)	23 (16.3)
500+	15 (11.1)	17 (11.6)	32 (11.4)	38 (55.9)	41 (56.2)	79 (56.0)
Missing	11	10	21	46	39	85
Ever diagnosed with TB						
Yes	9 (6.2)	9 (5.8)	18 (6.0)	7 (6.1)	6 (5.4)	13 (5.8)
Yes	50 (35.2)	36 (23.8)	86 (29.4)	41 (36.6)	23 (21.1)	64 (29.0)
Ever admitted to hospital						
Missing	4	5	9			
Pubertal delay						
Yes	8 (15.1)	19 (26.8)	27 (21.8)	5 (11.4)	14 (24.1)	19 (18.6)
Not eligible	93	85	178	70	54	124

At ART initiation, stunting was independently associated with older age, CD4 count < 200 cells/μl (OR = 1.29, 95% CI 1.05–1.58), TB history (OR = 3.41, 95% CI: 1.12–10.34) and previous hospital admission (OR = 1.77, 95%CI 1.02–3.09) (Table 2). Underweight was independently associated with older age, CD4 count< 200 (OR = 1.32, 95% CI: 1.07–1.62), male gender (OR = 1.95, 95%CI 1.12–3.57), and TB history (OR = 5.94, 95%CI: 1.76–20.08). Wasting was independently associated with older age, TB history (OR = 3.39, 95% CI: 1.14–10.07) and previous hospital admission. The odds of each outcome were similar for children aged 7–9 years as for those aged 9–11 years, and increased for children aged ≥12 years. Likelihood ratio tests confirmed that models with age as binary (7–11 years and > 12 years) fitted the data just as well as models with age in the original 4 categories.

**Table 2:** Association of factors with stunting, underweight and wasting at ART initiation

	Stunting (HAZ < -2)			Underweight (WAZ < -2)			Wasting (BAZ < -2)		
	Univariate	Multivariate (*N* = 272)		Univariate	Multivariate (*N* = 267)		Univariate	Multivariate (*N* = 289)	
	OR (95% CI)	OR (95% CI)	*p*-value	OR (95% CI)	OR (95% CI)	p-value	OR (95% CI)	OR (95% CI)	*p*-value
CD4 count									
≥200	1	1	0.01	1	1	0.01	1		
< 200	1.26 (1.04, 1.53)	1.29 (1.05, 1.58)		1.27 (1.05, 1.54)	1.32 (1.07, 1.62)		1.09 (0.86, 1.39)		
Age (years)									
6–8	1	1	0.05	1	1	< 0.001	1		0.006
9–11	0.76 (0.38–1.53)	0.95 (0.43–2.09)		0.44 (0.21, 0.89)	0.43 (0.19, 0.97)		1.59 (0.48, 5.24)	2.13 (0.54, 8.31)	
12–13	1.56 (0.76–3.23)	2.19 (0.98–4.90)		1.23 (0.60, 2.54)	1.46 (0.66, 3.23)		3.82 (1.19, 12.25)	6.02 (1.59, 22.72)	
14–17	1.69 (0.82–3.51)	2.19 (0.97–4.96)		1.76 (0.85, 3.64)	2.04 (0.91, 4.60)		4.21 (1.31, 13.54)	6.27 (1.66, 23.67)	
Sex									
Female	1	1	0.33	1	1	0.02	1	1	0.16
Male	1.21 (0.75, 1.94)	1.30 (0.77, 2.21)		1.87 (1.15, 3.04)	1.95 (1.12, 3.37)		1.59 (0.84, 3.02)	1.63 (0.82, 3.24)	
Orphan									
Both parents alive	1			1			1		
1 parent alive	0.91 (0.54, 1.55)			1.35 (0.79, 2.29)			1.39 (0.69, 2.80)		
Both dead	0.83 (0.43, 1.61)			0.81 (0.40, 1.63)			0.85(0.33, 2.20)		
Ever had TB									
No	1	1	0.03	1	1	0.01	1	1	0.03
Yes	3.18 (1.19, 8.46)	3.41 (1.12, 10.34)		2.90 (1.07, 7.87)	5.94 (1.76, 20.08)		3.10 (1.10, 8.71)	3.39 (1.14, 10.07)	
Hospital admission									
Never	1	1	0.04	1	1	0.09	1	1	0.04
Ever	1.81 (1.08, 3.04)	1.77 (1.02, 3.09)		1.97 (1.16, 3.33)	1.65 (0.93, 2.93)		1.96 (1.00, 3.83)	2.06 (1.02, 4.15)	

Pubertal delay was assessed in the 71 girls who passed their 13th birthday either before enrolment or during follow-up, and the 53 boys who reached age > 14 by end of follow-up. Pubertal delay was more common in girls than boys (26.8% versus 15.1%; *p* = 0.12). Over a third (35.2%) of adolescents with pubertal delay were stunted at ART initiation, compared to 12.7% of non-delayed adolescents of the same age. Pubertal delay was not associated with age at ART initiation or with CD4 count.

Mean HAZ was − 1.65 at ART initiation and remained at − 1.65 after 1 year (*p* = 0.85; Table 1). In contrast, there was strong evidence that WAZ increased over the first year of ART (mean − 1.59 at baseline and − 1.45 at follow-up; *p* = 0.007), as did BAZ (mean − 0.91 at baseline and − 0.70 at follow-up; *p* = 0.004).

### Spline models

**Fig. 2:**
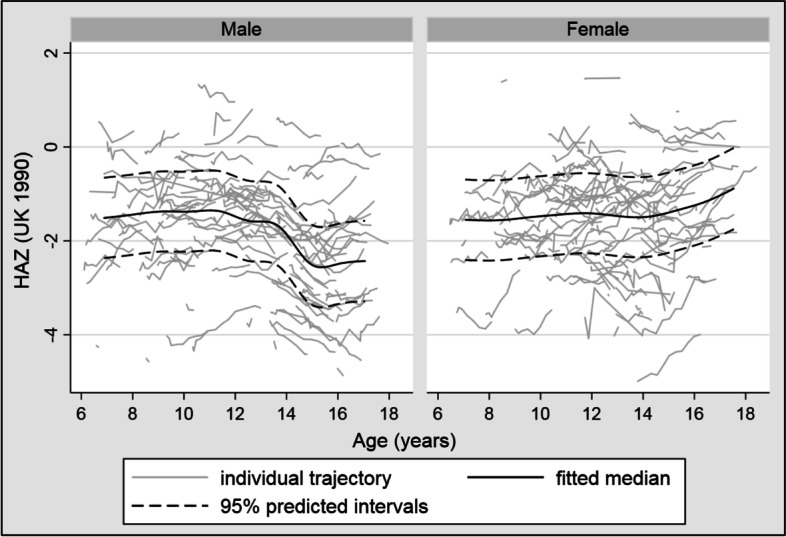
Restricted cubic spline model of HAZ over time by gender

Tables 3 and 4 shows HAZ as predicted by the model for all values of baseline CD4 count category, baseline HAZ category and gender, and for age in exact years from 6 to 18. In boys HAZ changed very little from aged 6 to 12, then decreased, reaching its lowest value at age 14–15, and then increased rapidly at ages 16–18. The biggest predictor of HAZ over time was HAZ at ART initiation (Table 3). In girls HAZ was the same as boys for ages 6–10 but from then on girls had higher HAZ than boys, with a much smaller dip at puberty (Table 4). The coefficients are presented in Table S1. The knots for the HAZ model were at ages 7.6, 10.6, 12.6, 14.5 and 16.5 years. Figure 2 shows the smoothed results from the median-spline plot.

**Table 3:** Predicted HAZ by age, CD4 count at ART initiation and HAZ at ART initiation for boys

Age (yrs)	CD4 at ART init.	< 200				200–349				350–499				500+			
	*HAZ at ART init.*	*<−3*	*− 3 to < − 2*	*− 2 to < − 1*	*> = − 1*	*<− 3*	*− 3 to < − 2*	*− 2 to < − 1*	*> = − 1*	*<− 3*	*− 3 to < − 2*	*− 2 to < − 1*	*> = − 1*	*<− 3*	*− 3 to < − 2*	*− 2 to < − 1*	*> = − 1*
6		− 3.71	− 2.47	− 1.51	− 0.44	− 3.76	− 2.52	− 1.55	− 0.49	− 3.55	− 2.32	− 1.35	− 0.28	− 3.63	− 2.39	− 1.42	− 0.36
7		− 3.72	− 2.48	− 1.52	− 0.45	− 3.77	−2.53	− 1.56	− 0.50	− 3.56	− 2.32	− 1.36	− 0.29	− 3.64	− 2.40	− 1.43	− 0.37
8		− 3.73	− 2.49	− 1.53	− 0.46	− 3.78	− 2.54	− 1.57	− 0.51	− 3.57	− 2.33	− 1.37	− 0.30	− 3.65	− 2.41	− 1.44	− 0.38
9		− 3.74	− 2.50	− 1.54	− 0.47	− 3.78	− 2.55	− 1.58	− 0.52	− 3.58	− 2.34	− 1.38	− 0.31	− 3.66	− 2.42	− 1.45	− 0.39
10		− 3.75	− 2.51	− 1.54	− 0.48	− 3.79	− 2.55	− 1.59	− 0.52	− 3.59	− 2.35	− 1.38	− 0.32	− 3.66	− 2.42	− 1.46	− 0.39
11		− 3.74	− 2.50	− 1.54	− 0.47	− 3.79	− 2.55	− 1.58	− 0.52	− 3.58	− 2.34	− 1.38	− 0.31	− 3.66	− 2.42	− 1.45	− 0.39
12		−3.75	− 2.51	− 1.55	− 0.48	− 3.79	− 2.56	− 1.59	− 0.52	− 3.59	− 2.35	− 1.39	− 0.32	− 3.66	− 2.43	− 1.46	− 0.40
13		−3.81	− 2.57	− 1.61	− 0.54	−3.86	− 2.62	− 1.65	− 0.59	− 3.65	−2.41	− 1.45	− 0.38	− 3.73	−2.49	− 1.52	− 0.46
14		−3.90	−2.66	− 1.69	− 0.63	− 3.94	−2.70	− 1.73	− 0.67	− 3.74	− 2.50	− 1.53	− 0.47	− 3.81	− 2.57	−1.60	− 0.54
15		−3.89	− 2.65	−1.68	− 0.62	− 3.93	− 2.69	− 1.73	− 0.66	− 3.73	− 2.49	− 1.52	− 0.46	− 3.80	− 2.56	− 1.60	− 0.53
16		− 3.76	− 2.52	− 1.55	− 0.49	− 3.80	− 2.56	−1.60	− 0.53	− 3.60	− 2.36	− 1.39	− 0.33	− 3.67	− 2.43	− 1.47	− 0.40
17		− 3.59	− 2.35	− 1.38	− 0.32	− 3.63	−2.39	− 1.43	− 0.36	− 3.43	− 2.19	− 1.22	− 0.16	− 3.50	− 2.26	− 1.30	− 0.23
18		− 3.41	− 2.18	− 1.21	− 0.14	− 3.46	− 2.22	− 1.25	− 0.19	− 3.25	− 2.02	− 1.05	0.02	− 3.33	−2.09	− 1.12	− 0.06

**Table 4:** Predicted HAZ by age, CD4 count at ART initiation and HAZ at ART initiation for girls

Age (yrs)	CD4 at ART init.	< 200				200–349				350–499				500+			
	*HAZ at ART init.*	*<− 3*	*− 3 to < − 2*	*− 2 to < − 1*	*> = − 1*	*<− 3*	*− 3 to < − 2*	*− 2 to < − 1*	*> = − 1*	*<− 3*	*− 3 to < − 2*	*− 2 to < − 1*	*> = − 1*	*<− 3*	*− 3 to < − 2*	*−2 to < − 1*	*> = − 1*
6		− 3.70	−2.46	− 1.49	− 0.43	− 3.74	− 2.50	− 1.54	− 0.47	−3.54	− 2.30	− 1.33	− 0.27	− 3.61	− 2.37	− 1.41	− 0.34
7		− 3.71	− 2.47	− 1.50	− 0.44	− 3.75	− 2.51	− 1.54	−0.48	−3.55	− 2.31	− 1.34	−0.28	−3.62	− 2.38	− 1.41	− 0.35
8		− 3.71	− 2.47	− 1.51	− 0.44	−3.76	− 2.52	− 1.55	− 0.49	− 3.55	− 2.31	− 1.35	− 0.28	−3.63	− 2.39	− 1.42	− 0.36
9		− 3.72	− 2.48	− 1.51	− 0.45	−3.76	− 2.52	− 1.56	−0.49	− 3.56	− 2.32	− 1.35	−0.29	−3.63	− 2.40	− 1.43	− 0.36
10		− 3.73	− 2.49	− 1.52	− 0.46	−3.77	− 2.53	− 1.57	− 0.50	− 3.57	− 2.33	− 1.36	− 0.30	−3.64	− 2.40	− 1.44	− 0.37
11		− 3.67	− 2.43	− 1.46	− 0.40	−3.71	− 2.47	− 1.50	− 0.44	− 3.51	− 2.27	− 1.30	− 0.24	−3.58	− 2.34	− 1.38	− 0.31
12		− 3.63	− 2.39	− 1.42	− 0.36	−3.67	− 2.43	− 1.46	− 0.40	− 3.47	− 2.23	− 1.26	− 0.20	−3.54	− 2.30	− 1.33	− 0.27
13		− 3.64	− 2.40	− 1.44	− 0.37	−3.68	− 2.45	− 1.48	− 0.42	− 3.48	− 2.24	− 1.28	− 0.21	−3.56	− 2.32	− 1.35	− 0.29
14		− 3.69	− 2.45	− 1.48	− 0.42	−3.73	− 2.49	− 1.53	− 0.46	− 3.53	− 2.29	− 1.32	− 0.26	−3.60	− 2.37	− 1.40	− 0.33
15		− 3.66	− 2.42	− 1.45	− 0.39	−3.70	− 2.46	− 1.49	− 0.43	− 3.50	− 2.26	− 1.29	− 0.23	−3.57	− 2.33	− 1.36	− 0.30
16		− 3.50	− 2.26	− 1.29	− 0.23	− 3.54	− 2.30	− 1.34	− 0.27	−3.34	− 2.10	− 1.13	− 0.07	−3.41	− 2.17	− 1.21	− 0.14
17		−3.30	− 2.06	− 1.09	− 0.03	− 3.34	− 2.10	− 1.14	− 0.07	−3.14	− 1.90	− 0.93	0.13	− 3.21	− 1.97	− 1.01	0.06
18		− 3.10	− 1.86	− 0.90	0.17	−3.15	− 1.91	− 0.94	0.12	− 2.94	− 1.70	− 0.74	0.33	− 3.02	− 1.78	− 0.81	0.25

**Fig. 3:**
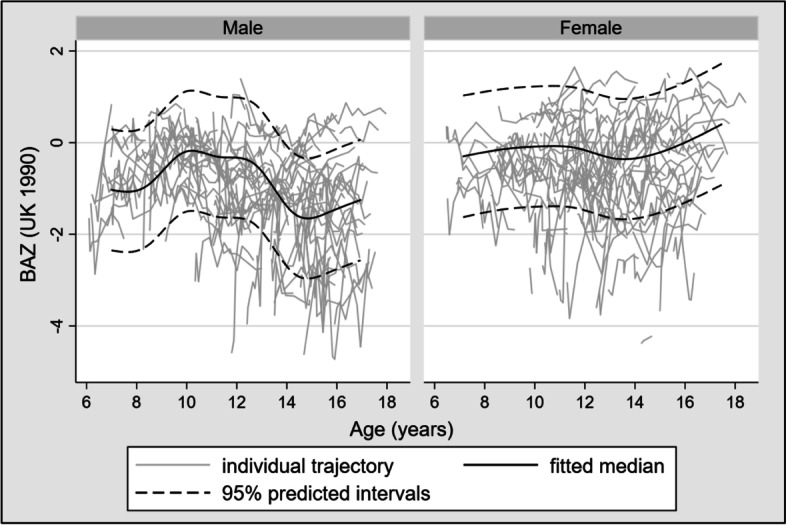
Restricted cubic spline model of BAZ over time by gender

Tables 5 and 6 shows predicted BAZ for all values of baseline CD4 count and BAZ categories, by gender, for all ages in years, predicted from the coefficients shown in Table S1. Similar to HAZ, BAZ among boys was stable in ages 6–10, then decreased to its lowest point at age 15, and then increased, and was closely associated with baseline BAZ (Table 5). In girls BAZ was lower than boys at age 6 but increased to age 10, then decreased until age 14 and then increased again. Overall, girls had higher BAZ than boys (Table 6). Knots were at 7.6, 10.6, 12.6, 14.5 and 16.5 years. The smoothed results from the median-spline plot are shown in Fig. 3.

**Table 5:** Predicted BAZ by age, CD4 count at ART initiation and BAZ at ART initiation for boys

Age (yrs)	CD4 at ART init.	< 200				200–349				350–499				500+			
	*BAZ at ART init.*	*<−3*	*−3 to < − 2*	*− 2 to < − 1*	*> = − 1*	*<− 3*	*− 3 to < − 2*	*−2 to < − 1*	*> = − 1*	*<− 3*	*− 3 to < − 2*	*− 2 to < − 1*	*> = − 1*	*<− 3*	*− 3 to < − 2*	*− 2 to < − 1*	*> = − 1*
6		− 2.36	− 1.75	− 1.03	0.06	−2.43	− 1.82	− 1.09	0.00	− 2.59	− 1.98	− 1.25	− 0.17	− 2.26	− 1.65	− 0.92	0.16
7		−2.37	− 1.76	− 1.03	0.05	− 2.43	− 1.82	− 1.09	− 0.01	−2.59	− 1.98	− 1.26	− 0.17	− 2.26	− 1.65	− 0.92	0.16
8		− 2.37	− 1.76	− 1.03	0.05	− 2.43	− 1.82	− 1.09	− 0.01	− 2.60	− 1.99	− 1.26	− 0.17	− 2.26	− 1.65	− 0.93	0.16
9		− 2.37	− 1.76	− 1.04	0.05	− 2.44	− 1.83	− 1.10	− 0.01	− 2.60	− 1.99	− 1.26	− 0.18	− 2.27	−1.66	− 0.93	0.15
10		− 2.38	−1.77	− 1.04	0.04	− 2.44	− 1.83	− 1.10	− 0.02	− 2.60	− 1.99	− 1.27	− 0.18	− 2.27	− 1.66	− 0.93	0.15
11		− 2.51	−1.90	− 1.17	− 0.09	− 2.57	− 1.96	−1.24	− 0.15	− 2.74	− 2.13	−1.40	− 0.32	− 2.41	− 1.80	− 1.07	0.02
12		− 2.64	− 2.03	− 1.30	− 0.22	− 2.70	− 2.09	− 1.37	− 0.28	− 2.87	− 2.26	− 1.53	− 0.45	− 2.54	− 1.93	− 1.20	− 0.11
13		−2.77	− 2.16	− 1.44	− 0.35	− 2.83	−2.22	− 1.50	− 0.41	− 3.00	− 2.39	− 1.66	− 0.58	− 2.67	− 2.06	− 1.33	− 0.25
14		−2.86	− 2.25	− 1.52	− 0.44	− 2.92	−2.31	− 1.58	− 0.50	− 3.08	− 2.47	− 1.75	− 0.66	− 2.75	− 2.14	− 1.41	− 0.33
15		− 2.84	−2.23	− 1.50	−0.42	− 2.90	− 2.29	− 1.56	−0.48	− 3.06	− 2.46	− 1.73	− 0.64	−2.73	− 2.12	− 1.39	−0.31
16		−2.73	− 2.12	− 1.40	−0.31	− 2.80	−2.19	− 1.46	−0.37	− 2.96	−2.35	− 1.62	− 0.54	−2.63	− 2.02	− 1.29	−0.21
17		−2.59	− 1.98	− 1.25	−0.17	− 2.65	− 2.04	− 1.32	− 0.23	− 2.82	− 2.21	− 1.48	− 0.40	−2.49	− 1.88	− 1.15	−0.06
18		−2.45	−1.84	−1.11	−0.03	−2.51	− 1.90	−1.17	− 0.09	−2.68	− 2.07	−1.34	− 0.25	−2.34	−1.73	− 1.01	0.08

**Table 6:** Predicted BAZ by age, CD4 count at ART initiation and BAZ at ART initiation for girls

Age (yrs)	CD4 at ART init.	< 200				200–349				350–499				500+			
	*BAZ at ART init.*	*<−3*	*− 3 to < − 2*	*−2 to < − 1*	*> = − 1*	*<− 3*	*− 3 to < − 2*	*−2 to < − 1*	*> = − 1*	*<− 3*	*− 3 to < − 2*	*−2 to < − 1*	*> = − 1*	*<− 3*	*− 3 to < − 2*	*−2 to < − 1*	*> = −1*
6		− 2.69	−2.08	−1.36	− 0.27	−2.75	− 2.14	− 1.42	− 0.33	−2.92	− 2.31	−1.58	− 0.50	−2.59	−1.98	− 1.25	− 0.17
7		−2.62	− 2.01	− 1.28	−0.19	−2.68	− 2.07	−1.34	−0.26	−2.84	− 2.23	− 1.51	− 0.42	−2.51	−1.90	− 1.17	−0.09
8		−2.54	−1.93	−1.20	−0.12	−2.60	− 1.99	−1.26	− 0.18	− 2.77	−2.16	−1.43	− 0.34	−2.43	−1.82	− 1.10	−0.01
9		−2.46	−1.85	−1.13	−0.04	−2.53	− 1.92	−1.19	− 0.10	− 2.69	−2.08	−1.35	− 0.27	−2.36	−1.75	− 1.02	0.06
10		−2.39	−1.78	−1.05	0.03	−2.45	−1.84	− 1.11	− 0.03	−2.61	− 2.00	−1.28	−0.19	−2.28	− 1.67	− 0.94	0.14
11		−2.44	−1.83	−1.10	−0.02	−2.50	− 1.89	−1.16	− 0.08	− 2.66	−2.05	−1.33	− 0.24	−2.33	−1.72	−0.99	0.09
12		−2.48	−1.87	−1.15	−0.06	−2.55	− 1.94	−1.21	− 0.12	− 2.71	−2.10	−1.37	− 0.29	−2.38	−1.77	− 1.04	0.04
13		−2.54	−1.93	−1.20	− 0.11	− 2.60	− 1.99	−1.26	−0.18	− 2.76	−2.15	−1.42	− 0.34	−2.43	−1.82	− 1.09	−0.01
14		−2.55	−1.94	−1.21	−0.13	−2.61	− 2.00	−1.27	− 0.19	−2.78	− 2.17	−1.44	− 0.35	−2.44	−1.83	− 1.11	−0.02
15		−2.47	−1.86	−1.13	−0.05	−2.53	− 1.92	−1.20	− 0.11	− 2.70	−2.09	−1.36	− 0.28	−2.37	−1.76	− 1.03	0.06
16		−2.32	−1.71	− 0.98	0.10	−2.38	−1.77	− 1.05	0.04	− 2.55	− 1.94	−1.21	− 0.13	−2.22	−1.61	−0.88	0.21
17		−2.14	−1.53	−0.80	0.28	−2.20	−1.59	−0.86	0.22	−2.36	−1.75	−1.03	0.06	−2.03	−1.42	−0.69	0.39
18		−1.95	−1.34	−0.62	0.47	−2.02	−1.41	−0.68	0.41	−2.18	−1.57	−0.84	0.24	−1.85	− 1.24	−0.51	0.57

## Discussion

Childhood stunting is associated with a host of adverse health and economic outcomes in adulthood [14]. Our study showed a high prevalence of growth failure among children and adolescents initiating ART at aged 8–16 years. Over a third (34.8%) of participants were severely stunted (HAZ < -3) or moderately stunted (HAZ between − 3 and − 2) at ART initiation, while 15.1% had low BMI-for-age. A multiregional study including 8737 adolescents aged 10–19 found higher prevalence of stunting (49.6%) and wasting (17.8%) at ART initiation [15]. The results in this paper are in agreement with a CIPHER cohort collaboration analysis of 20,939 perinatally infected and late-diagnosed adolescents from 46 countries [16]. In east and southern Africa, adolescents at ART initiation had high prevalence of stunting (51.2%) and wasting (13.9%), and girls showed improved HAZ with age but boys did not [16].

A history of TB was strongly associated with underweight, stunting and wasting, after adjustment for confounders. These participants had been treated for TB between 2 and 12 years prior to the study, suggesting long-lasting adverse effects of paediatric TB on growth outcomes among children living with HIV. Alternatively, TB history may indicate children with higher risk of poverty and food insecurity. The three growth outcomes also showed adverse effects of later initiation on ART. This finding is consistent with results from the ARROW trial in Uganda and Zimbabwe [17], where slower pubertal development was associated with late ART initiation. Older age at ART initiation is also associated with lower bone density in Zimbabwean adolescents [18]. Stunting and underweight were associated with low CD4 count but, surprisingly, wasting was not. Stunting and wasting were associated with a history of hospital admission. Some of the multifactorial causes of impaired growth such as infections, gastrointestinal illnesses and chronic inflammation [19] may have caused hospitalisation. Boys had almost twice the adjusted odds of being underweight compared to girls.

Catch-up growth in height following ART initiation has been shown consistently in children aged < 3 years, but not in older children. In this cohort, HAZ and BAZ of boys decreased to their lowest point at age 14–15 years, whereas girls experienced continued increase in HAZ and BAZ over age. Similarly, a multiregional study of 8737 adolescents aged 10–19 years using linear mixed models showed that HAZ in boys decreased to its lowest level at age 14 years and then increased until age 19 years, while HAZ of girls increased with age without the same dip [15]. A 2018 review [19] identified 3 other recent studies of exclusively older children with HIV in low-income settings. First, a cohort in Thailand and other Asian countries showed increased mean HAZ over 5 years on ART, among adolescents aged 10–19 at initiation. Secondly, in Ethiopia the prevalence of stunting among 5–10 years olds at ART initiation was 64% but decreased to < 20% after 2 years on treatment [20]. Finally, a cross-sectional study in Senegal found that children aged 2–9 years had a lower prevalence of stunting 2.9 years (median) post-initiation than at ART initiation, but the stunting prevalence in 10–16 year olds had remained unchanged [21]. Growth outcomes over time appear to be highly dependent on age at ART initiation, with younger adolescents more likely to benefit [15].

At ART initiation, 15.1% of male and 26.8% of female participants had clinically apparent pubertal delay. The results are consistent with a higher prevalence of subclinical delayed onset of pubertal growth spurt. Another study in Zimbabwe found that 8/127 (6%) of adolescents aged 14–16 with HIV and on ART had pubertal delay versus 0/132 of the same age without HIV who were recruited in schools [22]. The fact that late ART initiation is associated with poorer growth outcomes and delayed puberty [17] indicates that HIV infection may itself retard puberty, with ART reversing the effect.

The effects on growth seen here may not be entirely attributable to HIV. In The Gambia, Prentice et al showed that stunted, HIV negative adolescents had a delayed growth spurt but an extended growth period, with men in particular reaching their final height in their early twenties [23]. This suggests some connection between stunting in early childhood and late but prolonged growth, whether the stunting was caused by HIV, by nutritional deficiency (as in The Gambia), or by other causes. Notably, in the ARROW study in Uganda and Zimbabwe, adolescents on ART were still significantly increasing in height at Tanner stage 5 [17]. Normally, bone growth is curtailed by closure of the growth plates at approximately age 16 years in girls and 17 years in boys, before Tanner stage 5 is reached. The exact mechanism behind the process of epiphyseal fusion is unknown [24]. A multi-country study in Asia found the median HAZ of 18-year-olds with perinatally acquired HIV was − 1.4 for women and − 1.6 for men [25]. Height at 18 years was considered to be final height. However, in fact the adolescents might not have finished growing. At ART initiation (median 11.4 years) 55% of those with a height measurement were stunted, indicating risk factors for delayed and protracted growth spurt.

There is conflicting evidence on whether the effect of ART on growth outcomes may be independent from its effect on viral suppression. In cohorts of children in Uganda (aged 2–7 years) and the USA (aged 0–17 years), height and weight z-scores improved in the 2 years after ART initiation even when viral suppression was not achieved [26, 27]. However, a larger cohort of 1212 children found that height and weight gains were lessened in virologic non-responders [28].

The strengths of the study are that the sample is representative of newly diagnosed older children and adolescents, more than 95% of whom were living with perinatally acquired HIV. Anthropometry measurements were frequent and performed in a standardised manner. The 1990 UK growth reference curves may not be ideal for the population in Africa. However, a recent anthropometry survey of 729 healthy children aged 7–13 in Zimbabwe found that UK growth curves performed better than the WHO or CDC reference curves [29]. Our study had several limitations. The models do not represent repeated measures on the same individuals over a decade, but are the mean scores of 302 participants followed for a median of 16.6 months each. Catch-up growth in height may have become apparent with a longer follow-up on ART. Only 124 adolescents (40.7%) were old enough at any time during follow-up to be assessed for pubertal delay. We have not adjusted for length of time on ART, which could reduce the apparent effect of ART on growth outcomes. There may also be survival bias in our estimates, as children who have survived with untreated perinatally acquired HIV are taller than expected because of high mortality among those who were smaller. Survival bias would cause an underestimate of the effect of untreated HIV on growth. The protocol stipulated appointments every 3 months, but in practice they were arranged around participants’ availability. Multiple imputation to impute the missing visits was not used, because there was not enough regularity to identify gaps in the schedule. A small number of measurements (< 10) could not be used because the z-score was below − 5, even though they may have been correct values. Information on lifetime hospital admission was self-reported, prone to recall bias, and did not indicate number of or reason for admissions.

In conclusion, adolescent boys with perinatally acquired HIV and late diagnosis are particularly at risk of growth failure in puberty. These findings demonstrate the important of timely HIV diagnosis and early ART initiation in order to prevent long-term harmful effects.

## Supplementary Information

**Additional file 1: Table S1.** Results of restricted cubic spline models of height-for-age and BMI-for-age z-scores by age, with an interaction between gender and age.

## Data Availability

The datasets analysed during the current study are available in the LSHTM Data Compass repository 10.17037/DATA.00002469.

## References

[1] Rose AM, Hall CS, Martinez-Alier N (2014). Aetiology and management of malnutrition in HIV-positive children. Arch Dis Child.

[2] Achan J, Kakuru A, Ikilezi G, Mwangwa F, Plenty A, Charlebois E, Young S, Havlir D, Kamya M, Ruel T (2016). Growth recovery among HIV-infected children randomized to Lopinavir/ritonavir or NNRTI-based antiretroviral therapy. Pediatr Infect Dis J.

[3] World Health Organisation: consolidated guidelines on the use of antiretroviral drugs for treating and preventing HIV infection: recommendations for a public health approach (second edition). In. Geneva, Switzerland; 2016. 27466667

[4] Barlow-Mosha L, Musiime V, Davies M-A, Prendergast AJ, Musoke P, Siberry G, Penazzato M (2017). Universal antiretroviral therapy for HIV-infected children: a review of the benefits and risks to consider during implementation. J Int AIDS Soc.

[5] Simms V, Dauya E, Dakshina S, Bandason T, McHugh G, Munyati S, Chonzi P, Kranzer K, Ncube G, Masimirembwa C (2017). Community burden of undiagnosed HIV infection among adolescents in Zimbabwe following primary healthcare-based provider-initiated HIV testing and counselling: a cross-sectional survey. PLoS Med.

[6] UNAIDS (2019). UNAIDS data 2019.

[7] Slogrove AL, Schomaker M, Davies M-A, Williams P, Balkan S, Ben-Farhat J, Calles N, Chokephaibulkit K, Duff C, CIPHER Global Cohort Collaboration (2018). The epidemiology of adolescents living with perinatally acquired HIV: a cross-region global cohort analysis. PLoS Med.

[8] Jesson J, Koumakpai S, Diagne NR, Amorissani-Folquet M, Koueta F, Aka A, Lawson-Evi K, Dicko F, Kouakou K, Pety T (2015). Effect of age at antiretroviral therapy initiation on catch-up growth within the first 24 months among HIV-infected children in the IeDEA west African pediatric cohort. Pediatr Infect Dis J.

[9] Schomaker M, Leroy V, Wolfs T, Technau KG, Renner L, Judd A, Sawry S, Amorissani-Folquet M, Noguera-Julian A, Tanser F (2017). Optimal timing of antiretroviral treatment initiation in HIV-positive children and adolescents: a multiregional analysis from southern Africa, West Africa and Europe. Int J Epidemiol.

[10] Ferrand R, Meghji J, Kidia K, Dauya E, Bandason T, Mujuru H, Ncube G, Mungofa S, Kranzer K (2016). The effectiveness of routine opt-out HIV testing for children in Harare, Zimbabwe. JAIDS.

[11] McHugh G, Simms V, Dauya E, Bandason T, Chonzi P, Metaxa D, Munyati S, Nathoo K, Mujuru H, Kranzer K (2017). Clinical outcomes in children and adolescents initiating antiretroviral therapy in decentralized healthcare settings in Zimbabwe. J Int AIDS Soc.

[12] Cole TJ, Freeman JV, Preece MA (1998). British 1990 growth reference centiles for weight, height, body mass index and head circumference fitted by maximum penalized likelihood. Stat Med.

[13] Shepherd BE, Rebeiro PF, Caribbean C (2017). South America network for HIVe: brief report: assessing and interpreting the association between continuous covariates and outcomes in observational studies of HIV using splines. J Acquir Immune Defic Syndr.

[14] Prendergast AJ, Humphrey JH (2014). The stunting syndrome in developing countries. Paediatr Int Child Health.

[15] Jesson J, Schomaker M, Malasteste K, Wati DK, Kariminia A, Sylla M, Kouadio K, Sawry S, Mubiana-Mbewe M, Ayaya S (2019). Stunting and growth velocity of adolescents with perinatally acquired HIV: differential evolution for males and females. A multiregional analysis from the IeDEA global paediatric collaboration. J Int AIDS Soc.

[16] Jesson J, Crichton S, Quartagno M, Yotebieng M, Abrams EJ, Chokephaibulkit K, Le Coeur S, Ake-Assi MH, Patel K, Collaborative Initiative for Paediatric HIV Education Research Global Cohort Collaboration (2022). Growth and CD4 patterns of adolescents living with perinatally acquired HIV worldwide, a CIPHER cohort collaboration analysis. J Int AIDS Soc.

[17] Szubert AJ, Musiime V, Bwakura-Dangarembizi M, Nahirya-Ntege P, Kekitiinwa A, Gibb DM, Nathoo K, Prendergast AJ, Walker AS, Team AT (2015). Pubertal development in HIV-infected African children on first-line antiretroviral therapy. Aids.

[18] Gregson CL, Hartley A, Majonga E, McHugh G, Crabtree N, Rukuni R, Bandason T, Mukwasi-Kahari C, Ward KA, Mujuru H (2019). Older age at initiation of antiretroviral therapy predicts low bone mineral density in children with perinatally-infected HIV in Zimbabwe. Bone.

[19] Williams PL, Jesson J (2018). Growth and pubertal development in HIV-infected adolescents. Curr Opin HIV AIDS.

[20] Ebissa G, Deyessa N, Biadgilign S (2016). Impact of highly active antiretroviral therapy on nutritional and immunologic status in HIV-infected children in the low-income country of Ethiopia. Nutrition.

[21] Cames C, Pascal L, Diack A, Mbodj H, Ouattara B, Diagne NR, Diallo NF, Msellati P, Mbaye N, Sy Signate H (2017). Risk factors for growth retardation in HIV-infected Senegalese children on antiretroviral treatment: the ANRS 12279 MAGGSEN pediatric cohort study. Pediatr Infect Dis J.

[22] Rukuni R, Rehman AM, Mukwasi-Kahari C, Madanhire T, Kowo-Nyakoko F, McHugh G, Filteau S, Chipanga J, Simms V, Mujuru H (2021). Effect of HIV infection on growth and bone density in peripubertal children in the era of antiretroviral therapy: a cross-sectional study in Zimbabwe. Lancet Child Adolesc Health.

[23] Prentice AM, Ward KA, Goldberg GR, Jarjou LM, Moore SE, Fulford AJ, Prentice A (2013). Critical windows for nutritional interventions against stunting. Am J Clin Nutr.

[24] Shim KS (2015). Pubertal growth and epiphyseal fusion. Ann Pediatr Endocrinol Metab.

[25] Bunupuradah T, Kariminia A, Aurpibul L, Chokephaibulkit K, Hansudewechakul R, Lumbiganon P, Vonthanak S, Vibol U, Saghayam S, Nallusamy R (2016). Final height and associated factors in perinatally HIV-infected Asian adolescents. Pediatr Infect Dis J.

[26] Musoke PM, Mudiope P, Barlow-Mosha LN, Ajuna P, Bagenda D, Mubiru MM, Tylleskar T, Fowler MG (2010). Growth, immune and viral responses in HIV infected African children receiving highly active antiretroviral therapy: a prospective cohort study. BMC Pediatr.

[27] Nachman SA, Lindsey JC, Moye J, Stanley KE, Johnson GM, Krogstad PA, Wiznia AA (2005). Pediatric ACTGST: growth of human immunodeficiency virus-infected children receiving highly active antiretroviral therapy. Pediatr Infect Dis J.

[28] Guillen S, Ramos JT, Resino R, Bellon JM, Munoz MA (2007). Impact on weight and height with the use of HAART in HIV-infected children. Pediatr Infect Dis J.

[29] Madanhire T, Ferrand RA, Attia EF, Sibanda EN, Rusakaniko S, Rehman AM (2019). Validation of the global lung initiative 2012 multi-ethnic spirometric reference equations in healthy urban Zimbabwean 7-13 year-old school children: a cross-sectional observational study. BMC Pulm Med.

