# Supplementary material for: Growth improvement following antiretroviral therapy initiation in children with perinatally-acquired HIV diagnosed in older childhood in Zimbabwe: a prospective cohort study
Source: BMC Pediatr. 2022 Jul 25;22:446. doi: 10.1186/s12887-022-03466-0 (PMC9317209; doi:10.1186/s12887-022-03466-0)
Supplement: Supplementary file 1 — Additional file 1: Table S1. Results of restricted cubic spline models of height-for-age and BMI-for-age z-scores by age, with an interaction between gender and age. [file 12887_2022_3466_MOESM1_ESM.docx]

Table S1 (supplementary table): results of restricted cubic spline models of height-for-age and BMI-for-age z-scores by age, with an interaction between gender and age

|  |  | HAZ | | BAZ | |
| --- | --- | --- | --- | --- | --- |
|  |  | **Coefficient (95% CI)** | **p-value** | **Coefficient (95% CI)** | **p-value** |
| Gender | Female | 0.05 (-0.54, 0.63) | 0.874 | 1.21 (0.06, 2.36) | 0.039 |
| Age at ART initiation (years) |  | -0.05 (-0.12, 0.03) | 0.214 | -0.18 (-0.29, -0.07) |  |
| CD4 count at ART initiation | <200 | 0.08 (-0.13, 0.29) | 0.020 | -0.06 (-0.29, 0.17) | 0.306 |
|  | 200-349 | -0.07 (-0.28, 0.13) |  | -0.07 (-0.29, 0.14) |  |
|  | 350-499 | 0.17 (-0.04, 0.39) |  | -0.19 (-0.42, 0.03) |  |
| HAZ/BAZ at ART initiation | Less than -3 | -3.33 (-3.56, -3.10) | <0.001 | -2.62 (-2.95, -2.29) | <0.001 |
|  | -3 to <-2 | -2.12 (-2.30, -1.95) |  | -1.84 (-2.09, -1.59) |  |
|  | -2 to <-1 | -1.25 (-1.40, -1.09) |  | -1.07 (-1.22, -0.91) |  |
| Age (1) |  | 0.00 (-0.00, 0.01) | 0.072 | 0.01 (0.00, 0.02) | 0.004 |
| Age (2) |  | 0.02 (0.00, 0.03) | 0.010 | -0.08 (-0.11, -0.05) | <0.001 |
| Age (3) |  | -0.19 (-0.25, -0.13) | <0.001 | 0.40 (0.26, 0.55) | <0.001 |
| Age (4) |  | 0.42 (0.33, 0.52) | <0.001 | -0.62 (-0.86, -0.38) | <0.001 |
| Age (1) * female |  | -0.00 (-0.01, 0.00) | 0.519 | -0.01 (-0.03, -0.00) | 0.010 |
| Age (2) * female |  | 0.00 (-0.02, 0.02) | 0.885 | 0.10 (0.06, 0.14) | <0.001 |
| Age (3) * female |  | 0.09 (0.01, 0.18) | 0.028 | -0.45 (-0.65, -0.25) | <0.001 |
| Age (4) * female |  | -0.23 (-0.37, -0.09) | <0.001 | 0.66 (0.33, 1.00) | <0.001 |
| Constant |  | -0.69 (-1.57, 0.19) |  | -1.95 (-3.66, -0.24) |  |
